# Intensive Mindfulness Meditation Reduces Frequency and Burden of Migraine: An Unblinded Single-Arm Trial

**DOI:** 10.1007/s12671-023-02073-z

**Published:** 2023-01-24

**Authors:** Madhav Goyal, Jennifer A. Haythornthwaite, Sharat Jain, Barbara Lee Peterlin, Megha Mehrotra, David Levine, Jason D. Rosenberg, Mary Minges, David A. Seminowicz, Daniel E. Ford

**Affiliations:** 1 Department of Medicine, Johns Hopkins University School of Medicine, Baltimore, USA; 2 Center for Primary Care, NorthBay Healthcare, Vacaville, CA, USA; 3 Department of Psychiatry & Behavioral Sciences, Johns Hopkins University School of Medicine, Baltimore, USA; 4 Mid-Atlantic Vipassana Association, Claymont, DE, USA; 5 Neuroscience Institute, Penn Medicine Lancaster General Health, Lancaster, PA, USA; 6 Department of Epidemiology and Biostatistics, Univ of California, San Francisco, USA; 7 Department of Neurology, Mid-Atantic Permanente Medical Group, MD, Halethorpe, USA; 8 Department of Psychiatry and Behavioral Sciences, Montefiore Medical Center, Albert Einstein College of Medicine, New York, USA; 9 Department of Neural & Pain Sciences, School of Dentistry, Center to Advance Chronic Pain Research, University of Maryland Baltimore, Baltimore, USA

**Keywords:** Headache, Chronic, Episodic, Behavioral, Therapy

## Abstract

**Objectives:**

Preventing migraine headaches and improving the quality of life for patients with migraine remains a challenge. We hypothesized intensive meditation training would reduce the disease burden of migraine.

**Method:**

An unblinded trial was analyzed as a single cohort exposed to a silent 10-day Vipassana meditation retreat that included 100 hr of sitting meditation. Participants with chronic or episodic migraine were enrolled and followed for 1 year. The primary outcome was a change in mean monthly migraine days at 12 months from baseline. Secondary outcomes included headache frequency and intensity, acute medication use, work days missed, home meditation, sleep quality, general health, quality of life, migraine impact, positive and negative affect, perceived stress, mindfulness, and pain catastrophizing.

**Results:**

Three hundred people were screened and 58 (19%) agreed to participate and enrolled in the intensive meditation training. Forty-six participants with chronic migraine (≥ 15 headaches/month of which ≥ 8 were migraines) and 12 with episodic migraine (< 15 and ≥ 4 migraines/month) attended and 45 (78%) completed the retreat. At 12 months, the average migraine frequency was reduced by 2.7 days (from 16.6 at baseline) per 28 days (95%CI − 4.3, − 1.3) and headaches by 3.4 (20.1 at baseline) per 28 days (− 4.9, − 1.9). Fifty percent responder rate was 29% for migraine. Acute medication use dropped by an average of 2.2 days (− 3.9, − 0.5) per 28 days, and participants reported 2.3 fewer days (− 4.0, − 0.5) on which they reduced their activity due to migraines. The most striking and promising effects were in several secondary outcomes, including migraine-specific quality of life, pain catastrophizing, and perceived stress. The significant improvements observed immediately following the intervention were sustained at 12 months follow-up.

**Conclusions:**

Training in Vipassana meditation via a 10-day retreat may reduce the frequency and burden of migraine.

**Preregistration:**

ClinicalTrials.gov: NCT00663585.

Migraine occurs in 12% of the population in western countries, with women experiencing a disproportionate disease burden (Lipton et al., 2007). Two percent of the population suffers from chronic migraine in which they have a headache on more than 50% of days (Manack et al., 2011). While stress is thought to be an important factor in triggering migraine headaches, its role is complex and incompletely understood (Martin, 2016). In a large population-based study of over 5000 participants reporting headaches quarterly for 2 years, every 10-point increase in perceived stress was associated with a 4% increase in migraine headaches per month (Schramm et al., 2015). Prospective electronic diary studies have indicated that changes in stress (i.e., both increases and reductions) may precipitate migraines (Lipton et al., 2014; Schoonman et al., 2007).

Cognitive and affective mental processes augment the experience of pain, including pain catastrophizing, which is characterized by rumination about pain, magnifying its consequences, and feeling helpless to alleviate pain (Sullivan et al., 1995). Higher levels of pain catastrophizing correlate with headache attacks of higher frequency, duration, and impact (Bond et al., 2015; Holroyd et al., 2007). Interventions that reduce catastrophizing may have the potential to mitigate the impact of pain.

Those with chronic migraine who visit headache clinics have often tried and “failed” multiple pharmacologic therapies. FDA-approved medications for migraine prophylaxis are typically tested in those with episodic migraine and only show moderate efficacy. Systematic reviews of preventive agents such as topiramate have reported reductions of 0.4 to 1.5 headaches per month in episodic migraine when comparing the drug with a placebo. They have also reported 20–40% fifty percent responder rates (proportion of participants with at least a 50% reduction in migraine frequency) with these medicines (Jackson et al., 2015; Shamliyan et al., 2013). Placebo effects alone often yield a reduction of one headache per month (Jackson et al., 2015). In all, while pharmacologic therapies are a cornerstone of current therapy, clinical trials of existing pharmacologic treatments support a reduction of only one to two migraines per month beyond placebo effects. These comparisons with placebo are true of the newer calcitonin gene-related peptide (CGRP) antagonists as well (Detke et al., 2018; Dodick et al., 2018; Reuter et al., 2018; Silberstein et al., 2017; Stauffer et al., 2018; Tepper et al., 2017). Given the relatively small absolute reductions observed, preventing migraine attacks remains a significant challenge for clinicians.

Further complicating the application of these data to the clinic, trials tend to have many exclusion criteria which likely inflate the benefit these medications would have in real-world settings. Several trials were limited by large numbers of withdrawals, most of which were due to adverse effects from the medication or a lack of efficacy (Brandes et al., 2004; Diener et al., 2004; Silberstein et al., 2007). The vast majority of trials were limited to short-term follow-up, providing little information about what happens beyond 2–6 months (Jackson et al., 2015; Loder & Rizzoli, 2018; Shamliyan et al., 2013).

Behavioral therapies have shown mixed results, with older reviews indicating headache reductions comparable to pharmacotherapy (Goslin et al., 1999). Recent reviews suggest a benefit in improving quality of life rather than a substantial reduction of headache frequency (Harris et al., 2015; Probyn et al., 2017; Wells et al., 2020). Meditation programs have moderate strength of evidence to reduce pain complaints but are understudied for headache disorders (Goyal et al., 2014). A recent randomized, controlled trial of meditation among episodic migraine patients showed 1.5 reduced headache days at 20 weeks compared to an active control. These reductions were maintained for 1 year, although no longer statistically different from the active control (Seminowicz et al., 2020). Existing trials of meditation for headaches tend to use relatively short exposures to training on the order of 5–30 hr over 2 to 4 months (Grazzi et al., 2017; Seminowicz et al., 2020; Seng et al., 2019; Simshauser et al., 2020).

We sought to observe whether intensive training in meditation followed by long-term daily practice could have measurable and enduring effects on migraine frequency, intensity, and quality of life. Vipassana meditation is taught at a silent 10-day retreat and remains one of the most intensive forms of standardized meditation training (Vipassana website, 2022). It is widely available worldwide, with no charge to attend training. Enrollees participate in up to 10 hr of sitting meditation per day (Hart, 2005; Vipassana Introduction to the Technique, 2022). The retreat is designed to minimize any distractions in order to allow students to maximally focus on developing mind–body skills. One skill that is cultivated is how to objectively observe mental phenomena and bodily sensations by becoming aware of and reducing one’s reactions to these stimuli. Due to the nature of this training, we hypothesized that Vipassana meditation would reduce pain catastrophizing and promote coping with stress, leading to reduction of migraine and enhancement of general health and quality of life. Specifically, we hypothesized that training in meditation would lead to reductions in headache frequency, intensity, 50% responder rate, acute medication use, work days missed, perceived stress, and pain catastrophizing. We also hypothesized it would lead to enhancement of sleep quality, quality of life, positive affect, and mindfulness.

## Method

### Participants

Participants were recruited from local headache and neurology clinics and community/web advertisements. Participants fulfilled the International Headache Society 2^nd^ edition criteria for migraines (Penzien et al., 2005). Participants enrolling in the trial had to report at least 15 headache days per month of which at least eight were migraines (fulfilling the definition for “chronic migraine”), while episodic migraine participants had to report at least 4 and less than 15 migraines per month. Exclusion criteria included anything that might interfere with participating in a 10-day course: severe depression or anxiety, panic attacks, psychosis, dementia, active alcoholism, or use of illicit drugs within the last 3 months; already completed a 10-day course or actively involved in another form of meditation over the past 6 months; women who were pregnant or planning pregnancy during the trial period, or lactating (Penzien et al., 2005). Participants could continue to use their prophylactic or acute medications throughout the study. Table 1 provides descriptive statistics for all participants. Based on a reduction of two migraines per month, assuming a standard deviation of three migraines per month in each group and alpha 0.05 and power of 80%, we estimated needing 36 participants per arm (committed vs not committed). The trial did not achieve its target sample size.

### Procedure

Participants were screened by phone and then attended in-person interviews to discuss the rigors and requirements of the meditation retreat (Vipassana website, 2022). Participants completed an independent online application to the Vipassana course of their choice and notified us upon acceptance. Local 10-day meditation retreats were held semi-annually or a non-local retreat could be selected if that was more convenient. All Vipassana retreats are standardized in terms of schedule, instruction, and practice (Vipassana website, 2022).

After signing informed consent, participants completed a baseline survey and at least 28 days of online daily headache diaries to finalize eligibility. For the randomized trial, chronic migraine participants were randomized on the last day of the 10-day retreat to either a commitment group or a no-commitment group. We hypothesized that the commitment group would meditate more regularly and that regular meditation would lead to positive outcomes over a 1-year period. Randomization was generated via an online random number generator by one researcher and allocation was sealed in numbered privacy envelopes. A second researcher held the envelopes and revealed allocation over the phone at the time of randomization. Those in the commitment group committed to meditate on a mutually agreed amount daily for the next 12 months. We asked that the amount be substantive, at least a half hour per day if possible. This discussion and commitment were not made with those in the no-commitment group. We also recruited participants with episodic migraine who attended the retreat and were not randomized. All participants were followed for 1 year after completing the retreat. All episodic migraine participants made a commitment on the last day of the 10-day retreat to meditate on a mutually agreed amount daily for the next 12 months. We included episodic migraine participants because we wanted to assess the response to meditation across the spectrum of migraine frequency.

The randomized trial failed to yield group differences in meditation frequency or outcomes. Therefore, in addition to the null results of the randomized trial, we report a pre-post analysis of the entire cohort. For the pre-post analysis, we hypothesized that we would see reductions in migraine frequency as well as increases in quality of life measures from baseline to 1 year following the retreat. While patients with migraines are traditionally dichotomized into episodic or chronic categories, the acute and prophylactic treatments used for both categories are largely the same in clinical practice. The reductions in headache frequency documented in the migraine literature using placebo controls are the same for both categories, with about one to two migraines per month. Thus, we decided to combine both groups in the pre-post analyses.

### Measures

Participants completed daily headache diaries for 12 months following the retreat. These were accessed from a computer or smartphone and took about 2–3 min to complete. Diaries included up to 24 questions depending on whether they had a headache or not on the previous day. If they had a headache, questions assessed headache characteristics, whether they had to miss work, use of acute medications, and sleep length and quality. The post-retreat diaries contained additional questions asking whether they sat to meditate and the total time they meditated. Survey Monkey was the survey tool used, and a daily reminder was sent to their email address to fill out the diary. The primary outcome was migraine frequency per 28 days. Secondary outcomes included headache frequency per 28 days, intensity, 50% responder rate (proportion of participants experiencing at least a 50% reduction in their migraine frequency), acute medication use, work days missed, home meditation, and sleep quality. Participants completed additional assessments at 2 weeks and 3, 6, and 12 months, which are additional secondary outcomes. These self-reported assessments were completed online at home and included the following.

#### General Health Questionnaire 28

The General Health Questionnaire 28 (GHQ28) is a 28-item instrument that assesses psychological well-being in four domains: somatic complaints, anxiety/insomnia, social dysfunction, and depression. Participants were instructed to rate statements on how the have felt over the past few weeks (e.g., “Have you recently been thinking of yourself as a worthless person?”) on a 4-point Likert scale (1 = *Not at all* to 4 = *Much more than usual*). It also gives an overall psychological well-being score (Goldberg & Hillier, 1979). Test–retest reliability has been reported to be high (0.78 to 0.9) as well as high internal consistency (Cronbach’s α 0.90) (Failde et al., 2000; Robinson & Price, 1982). The GHQ28 also correlates well with other measures of depression (Robinson & Price, 1982). In the current sample, the scale demonstrated good internal consistency and reliability (Cronbach’s *α* = 0.92, McDonald’s *ω* = 0.92).

#### Migraine-Specific Quality of Life

The Migraine-Specific Quality of Life (MSQ) is a 14-item instrument designed to measure the change over time in health-related quality of life for migraine patients. It consists of three dimensions that are affected by migraine: limitations to the performance of daily activities, interruptions to the performance of daily activities, and frustration/helplessness due to migraine. Sample statements include, “In the past 4 weeks, how often have migraines LIMITED the number of days you have felt energetic?” and “In the past 4 weeks, how often have you had to CANCEL work or daily activities because you had a migraine?” Participants rated these statements on a six-point scale (1 = *All of the time* to 6 = *none of the time*). The MSQ subscales have demonstrated good reliability (Cronbach’s α 0.86 to 0.96) and construct validity (Bagley et al., 2012; Martin et al., 2000; Speck et al., 2021). In the current sample, the scale demonstrated good internal consistency and reliability for all three subscales (*α* = 0.82 to 0.96, *ω* = 0.83 to 0.96).

#### Migraine Disability Assessment Test

The Migraine Disability Assessment Test (MIDAS) is a five-item instrument designed to measure headache-related disability suffered over the prior three months and two additional questions quantifying the frequency and severity of headaches over the prior three months. It asks participants to report the number of days of disability (e.g., “On how many days in the last 3 months did you miss work or school because of your headaches?”). It has shown good test–retest reliability (0.8), internal consistency (Cronbach’s *α* 0.76), and validation compared with a 90-day headache diary (Stewart et al., 2001). In the current sample, the scale demonstrated good internal consistency (*α* = 0.74) and poor reliability (*ω* = 0.55).

#### Positive and Negative Affect Schedule

The Positive and Negative Affect Schedule (PANAS) consists of two 10-item scales to measure positive and negative affect. Positive affect reflects the extent to which a person feels enthusiastic, active, and alert. Negative affect reflects subjective distress, anger, and fear. It has shown adequate test–retest reliability (0.68 to 0.71) for each of the subscales, high internal consistency (Cronbach’s *α* 0.87 to 0.88) and good convergent and discriminant validity (Watson et al., 1988). In the current sample, both subscales demonstrated good internal consistency and reliability (*α* = 0.93 to 0.94, *ω* = 0.93 to 0.94).

#### Perceived Stress Scale (4-Item Version)

The 4-item Perceived Stress Scale (PSS-4) is derived from the 14-item PSS, one of the most widely used instruments for measuring the perception of stress (Cohen et al., 1983). The abbreviated version was used to reduce the burden on our participants. The questions (e.g., “In the last month, how often have you felt that you were unable to control the important things in your life?) are rated on a 5-point scale (1 = *Never*; 5 = *Very often*). While there is a loss in reliability as compared with the original, the PSS-4 has demonstrated adequate internal consistency (Cronbach’s *α* range 0.77 to 0.82) and shown to be a valid measure of perceived stress in different populations (Karam et al., 2012; Mitchell et al., 2008; Warttig et al., 2013). In the current sample, the scale demonstrated good internal consistency and reliability (*α* = 0.74, *ω* = 0.76).

#### Freiburg Mindfulness Inventory

The Freiburg Mindfulness inventory (FMI) was developed on people attending Vipassana meditation retreats to measure mindfulness. It is a 14-item instrument with good internal consistency (Cronbach’s *α* 0.86) and has been shown to demonstrate an increase in mindfulness after a mindfulness retreat (Walach et al., 2006). Questions (e.g., “I accept unpleasant experiences”) are rated on a 4-point scale (1 = *Rarely* to 4 = *Almost always*). In the current sample, the scale demonstrated good internal consistency and reliability (*α* = 0.87, *ω* = 0.87).

#### Pain Catastrophizing Scale

Pain Catastrophizing Scale (PCS) is a negative cognitive-affective process that includes elements of magnification, helplessness, pessimism, and rumination (Buenaver et al., 2007). It is an important predictor of pain-related outcomes, and more frequent catastrophizing is reliably associated with heightened pain experience and emotional distress across many chronically painful conditions (Sullivan et al., 2001). The questions (e.g., “I worry all the time about whether the pain will end.”) are rated on a 5-point scale (1 = *Not at all*; 5 = *All the time*) The PCS is a 13-item instrument that provides an overall score, has good internal consistency (Cronbach’s *α* 0.87 to 0.95 for total score), and is one of the most widely used measures of pain-related catastrophic thinking (Osman et al., 2000; Sullivan et al., 1995). In the current sample, the scale demonstrated good internal consistency and reliability (*α* = 0.93, *ω* = 0.93).

### Data Analyses

A migraine day was defined as a calendar day in which headache pain lasted at least 4 hr and met criteria for migraine (with or without aura) or probable migraine (subtype in which only one migraine criterion is absent), or a day in which acute medication was used to treat headache of any duration (International Headache Society, 2020; Silberstein et al., 2017).

For the primary outcome of the trial, we used a generalized estimating equation (GEE) to compare the change in migraine frequency before and after the retreat between those assigned to the committed vs. not committed arm. In secondary analyses, we used GEE to conduct a pre-post analysis accounting for repeated measurements within individuals, evaluating the impact of the retreat on headache and migraine frequency, headache intensity and duration, medication use, and days of work missed due to headaches. For dichotomous outcomes, we used link logit with family binomial, and for continuous outcomes, we used link identity and family Gaussian, with robust standard errors to correct for the non-normal distribution of the continuous outcomes. We also used GEE across the post-intervention period to explore the extent to which any meditation during the past 24 hr (dichotomous) or the number of hours of meditation in the past 24 hr (continuous) were associated with the occurrence of headache or the intensity of headache. We assessed whether diary entries were systematically missing according to baseline demographics and prior headache or migraine severity, and we conducted sensitivity analyses to examine whether our results were sensitive to these missing data. Statistical tests were limited to two decimal places except where significant figures indicated fewer decimal places should be used.

## Results

**Fig. 1:**
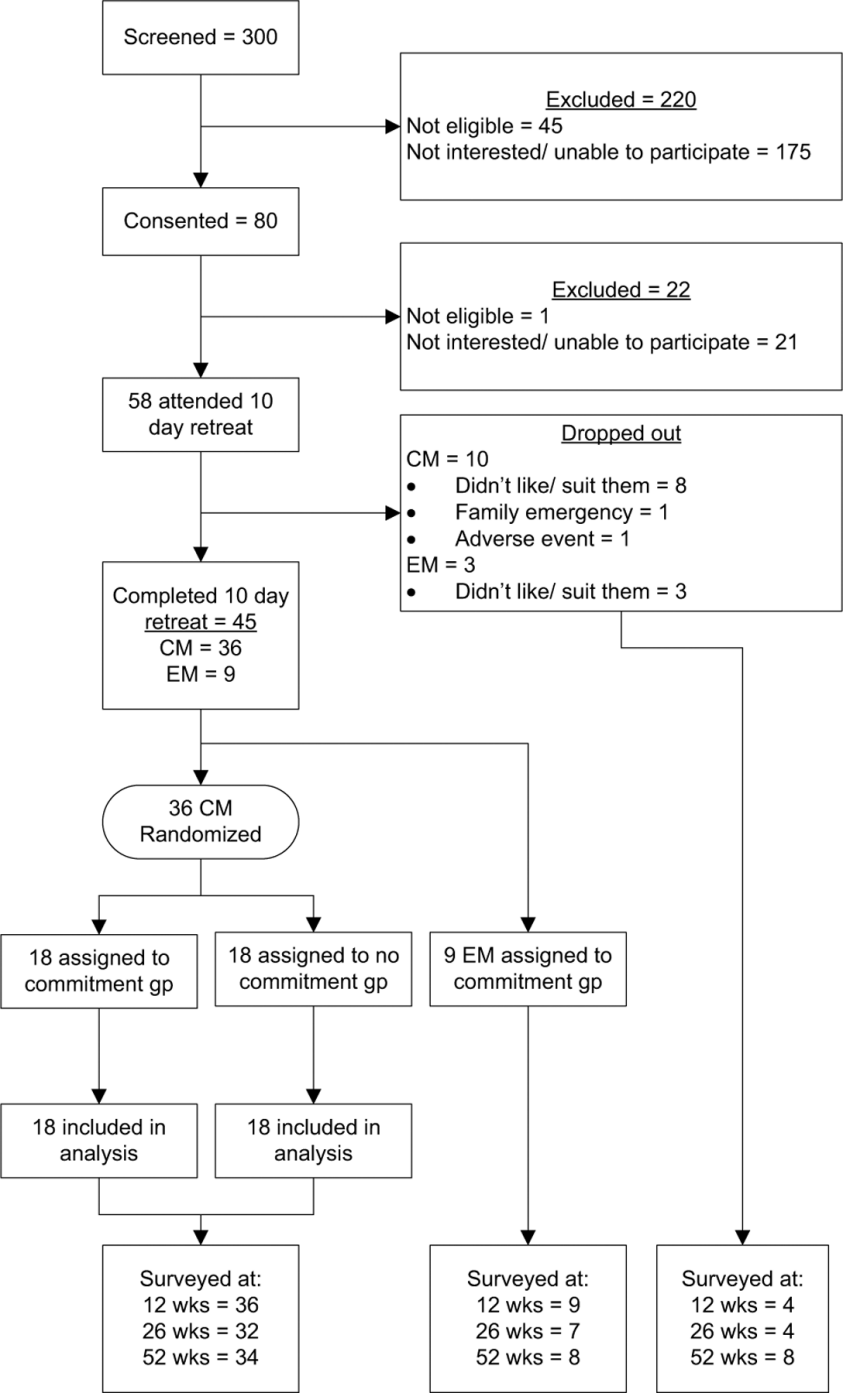
Recruitment and retention. CM, chronic migraine; EM, episodic migraine

Three hundred people were screened and 58 (19%) registered and attended one of the Vipassana retreats (Fig. 1). Thirteen individuals withdrew from the retreat prematurely; one individual left due to a family emergency, one left due to an adverse event (hallucination), and the remainder decided the retreat did not suit them. Forty-five (15% of the original 300) participants, 36 chronic migraine and nine episodic migraine, completed the retreat with follow-up over the subsequent 12 months. Those who finished the retreat completed 81% of the daily headache diaries, which were used in calculating the primary outcome. Those who did not finish the retreat completed 28%.

The majority of completers were white females (mean age of 47 years), holding at least a bachelor’s degree and earning greater than US$ 50,000 (Table 1), typically suffering from migraines for 17 years. The 13 non-completers were slightly younger and reported overall higher severity and impact of headaches. Of 38 completers who responded to open-ended questions about previous treatments for migraine, 21 (55%) indicated they had been treated at three or more clinics or hospitalized as inpatients for their migraines, 22 (58%) indicated they had tried many different therapies including nerve blocks or surgeries. Twenty (53%) indicated that no previous therapy had reliably helped them. On average completers meditated for 100.0 days (*SD* 109.0) out of the year, and the average amount of meditation done was 35.7 min per day (*SD* 50.0) on days that they meditated. Thirteen of 45 individuals meditated at least half an hour daily for more than 90 days, and six meditated at least half an hour daily for more than 180 days of the 1-year follow-up.

The intervention of asking participants to commit to a minimum amount of daily meditation had little effect on their meditation practice and did not yield greater practice. The average amount of meditation reported by the commitment group was 33.8 min/day compared with 34.0 min/day by the noncommitment group (*p* = 0.82). Asking participants to commit to meditate had no effect on migraine frequency (− 2.9 days from baseline to follow-up in the commitment group compared with − 4.1 days in the noncommitment group, *p* = 0.40).

### Effects of Intensive Training in the Cohort

Primary endpoint: Among participants who completed the retreat (Table 2), including both chronic migraine and episodic migraine participants, migraine frequency was reduced by an average of 2.7 days per 28 days, 95% Confidence Interval (CI) (− 4.3, − 1.3).

Secondary endpoints: During the 1 year of follow-up, we observed an average reduction of 3.4 headache days per 28 days, 95%CI (− 4.9, − 1.9), with a 0.7 (− 0.9, − 0.4) reduction in headache intensity. The 50% responder rates at 3 and 12 months were 31% and 29% for migraine, and 27% and 20% for headache. Acute medication use decreased by an average of 2.2 days (− 3.9, − 0.5) per 28 days and participants reported 2.3 fewer days (− 4.0, − 0.5) on which they reduced their activity due to migraines. There were no changes observed in duration of headache, missed work days or sleep duration or quality. MIDAS scores declined by 40% from baseline to 12 months (Table 3). Migraine specific quality of life improved in all 3 domains (Restrictive, Preventive, Emotional) by 20–40% over the 12-month follow-up (*p* < 0.01). Pain Catastrophizing Scale scores improved by about 50% at the 12-month follow-up (*p* < 0.01). There were variable improvements in other outcomes, with a dip in scores at six months for most outcomes. We did not observe any association between the amount of daily meditation and reduction in headache frequency (odds ratio [OR] = 1.01 [0.92, 1.11]) or severity (− 0.15 reduction on a 0–10 scale [− 0.42, 0.12]) over the 1-year follow-up. Among the 13 retreat non-completers, no consistent changes over time in any of the outcomes were observed.

#### Sensitivity Analysis

Missing data on the daily headache diaries were analyzed for any systematic patterns associated with our outcomes. We evaluated whether having a headache on one day or two sequential days, or intensity of headache on those days, was associated with missingness on the subsequent day. We also assessed whether the randomization group was associated with missingness. We did not find any such associations. We conducted sensitivity analyses by assuming (1) that everyone had a headache on the day of missing data, (2) no one had a headache on the day of missing data, and (3) no one had a headache on the day of missing data pre-retreat and everyone had a headache on the day of missing data post-retreat. In all of these scenarios, we found a statistically significant reduction post-retreat in headache frequency.

### Safety

One adverse event occurred in which a participant with no prior history of psychosis who was taking protriptyline for migraines had a hallucination during the retreat. This participant left the retreat mid-way and did not report any further hallucinations on follow-up.

## Discussion

We conducted a study on the outcomes of people with migraine who completed intensive meditation training that modeled treatment in a real-world clinical setting, allowing individuals to choose whether to participate in an intensive, silent, 10-day training retreat. We found that full participation in the retreat was associated with a reduction in migraine and headache frequency and stress for up to 1 year following the training compared to baseline. Despite the demands of the 10-day training, our drop-out rate (22%) was similar to that observed in trials of both pharmacological and non-pharmacological treatments. Completers also reported a reduction in acute medication use, and multiple psychological factors improved. For example, participants experienced a significant improvement in pain catastrophizing, perceived stress, and ability to perform daily activities. These improvements were maintained at 1 year.

We hypothesized that daily practice of meditation following the 10-day training would lead to improved long-term outcomes. We did not observe any association between the amount of daily meditation and headache frequency or severity over the 1-year follow-up, although we had limited power to find a dose–response relationship. We did see reductions in perceived stress and pain catastrophizing which may contribute to reduced migraines. These reductions, along with statistically significant 20–40% improvements in quality of life, were substantial. Although the amount of daily meditation was not large, it may be that the initial 10-day retreat had a lasting effect, or that the combination of the initial retreat followed by small doses of meditation had this effect.

Very few trials exist that evaluate the effects of mindfulness programs on headaches. Two trials evaluated short-term outcomes at 8 to 16 weeks and found the mindfulness arm reduced headaches by an average of 1.3 to 1.5 per month, while the control arm showed a reduction of about 1.0 per month (Seng et al., 2019; Simshauser et al., 2020). Two trials evaluated longer term outcomes. Wells et. al. (2021) randomized 96 people to MBSR vs headache education and found the MBSR showed an average of 2.2 fewer migraines at 36 weeks compared to baseline, but the education arm showed 2.7 fewer migraines. The trial by Seminowicz et al. (2020) compared an enhanced MBSR course to stress management for headache arm and found 1.2 fewer migraines at 52 weeks as compared with 0.1 fewer migraines in the stress management arm (Seminowicz et al., 2020).

In these long-term trials, two issues likely account for the inconsistencies. The first issue is the duration and focus of training. The trial by Seminowicz et al. (2020) enhanced the typical MBSR training by lengthening the course and focusing the training on migraine. The MBSR + group showed a rapid and significant reduction in headaches that were maintained at 52 weeks. The Wells trial used a standard MBSR format. The second issue is the differential response in the control groups. Wells et al. (2021) found headache education to be effective immediately, whereas Seminowicz et al. (2020) found stress management provided modest, delayed effects at 52 weeks. Depending on the nature of the education control, these control groups may have significant treatment responses and may have more durable effects than pill placebos. Due to the practical difficulties of running a placebo arm that is comparable to a 10-day retreat, only a handful of Vipassana studies are available and mostly measure pre-post outcomes. These studies show improvements in psychological health including anxiety, depression, stress, and well-being (Al-Hussaini et al., 2001; Khoury et al., 2017; Maruthai et al., 2016; Montero-Marin et al., 2016). They also show improvements in substance abuse, psychosocial functioning, and decreased mood disturbances among incarcerated populations (Bowen et al., 2006; Perelman et al., 2012; Ronel et al., 2013) but none of these measured headaches.

As with most interventions for migraine, meditation may not be for everyone. Although the use of meditation has more than tripled in the USA in the past decade, participants who chose to attend the retreat (19%) represent a small portion of the migraine patients who expressed interest in our study (National Center for Complementary & Integrative Health, 2019). Our recruitment goals were not met because local retreats occurred only twice per year requiring long delays for some interested participants; the logistics of home and work responsibilities limited others’ ability to be away for 10 days; and some expressed anxiety about spending 10 days in silence and isolated from family and friends. While this recruitment may appear exclusive, both pharmacologic and non-pharmacologic studies often have comparable enrollment rates. It is not unusual in headache trials to exclude patients not having a therapeutic response to two or more preventive regimens; using medicines such as B-blockers, antidepressants, antiepileptics, calcium channel blockers, or non-steroidal anti-inflammatories; overusing acute medications; using herbal preparations such as fever-few or St. John’s wort; or having continuous pain for the past month, among others (Brandes et al., 2004; Silberstein et al., 2007, 2017; Tepper et al., 2017). Our study did not apply any such exclusions, but rather allowed the real-life motivations and factors that affect an individual’s ability and willingness to drive participation in our study.

Finally, while there is limited data on adverse events, meditation is generally not recommended for people with psychosis. Medications such as protriptyline, in which hallucinations are a possible side effect, might be best avoided during an intensive meditation retreat. More data are needed to understand these possible interactions.

## Limitations and Future Research

In the absence of a comparison group, it is possible that the reductions in migraine frequency reflect regression to the mean. Regression to the mean may account for the reduction because migraine headaches vary in intensity and frequency over time. The individual may have agreed to join the study when their headaches were especially bad and the reduction in severity occurred due to random variation. We asked research participants about their history of migraines and did not find that their pattern of headaches at the time of enrollment was worse than their usual state. As outcome measures were obtained through online systems and independent from contact with investigators, bias in reporting outcomes was minimized and the durability of the reduction in headaches over a 1-year period adds credibility to their validity. It is also possible that the retreat helped participants to reduce their use of medications, and this in turn reduced their headaches in the long term. This is a complex issue as most participants had seen several providers and tried multiple treatments that were ineffective for them over many years. These issues warrant further investigation.

While it is possible that the results can be explained by placebo effects, a recent meta-analysis of prophylactic treatments for migraines that evaluated the placebo effect suggests otherwise. Analyzing data from 78 randomized trials, significant reductions in migraine frequency in the placebo group were only seen at 4 to 12 weeks. By 16, 20, and 24 weeks the number of headaches experienced by patients given a placebo increased back to baseline rates (Jackson et al., 2015). Since these placebo effects included a brief reduction of 1–2 headaches/month, the treatment effects we observed following intensive meditation training—an average headache reduction of approximately 3 headaches/month that endure for 12 months—likely surpass any placebo effect.

Common methods bias often occurs in self-reported measures when the predictor and criterion variables are measured in the same survey using a similar format (Podsakoff et al., 2012). Our primary outcome, migraine frequency, was measured by a daily headache diary using a yes/no response. These diaries also measured some of our secondary outcomes, including acute medication use, work days missed, home meditation, and sleep quality. Each of these used different response options. The responses to these scales in these diaries may be correlated due to being on the same survey. The items from the periodic surveys which were administered at 2 weeks and 3,6, and 12 months (e.g. Migraine Specific Quality of Life, Pain Catastrophizing Scale) were conducted separately from the daily headache diaries, largely used Likert rating scales, and although may be intercorrelated with each other are less likely to be correlated with the primary outcome (headache frequency measured in the daily diary).

Although it is not possible to tell if the reductions in migraine or headache frequency we observed are due to expectation effects, for patients and clinicians the important results may be that the absolute reductions and 50% responder rates are similar to what patients could expect using standard pharmacotherapy. For a subgroup of patients, this information may serve as a critical motivator in selecting an intervention that requires logistical planning and may evoke anxiety. Furthermore, this intervention is not specific for chronic or episodic migraine; it just needs to meet their threshold for the headaches being bothersome enough. Also helpful to patients is that medication can continue to be used concurrently with the training and practice of meditation as seen in this trial.

While this study was not intended to generalize to all patients with migraine, the results are relevant for those who have an interest in and flexibility to pursue this type of in-depth self-exploration. Many patients prefer “natural” or nonpharmacologic methods to mitigate the ongoing expense of pharmaceuticals (Cottrell et al., 2002). Others prefer to reduce pharmaceutical exposure and/or side effects, especially women during child-bearing years (Cottrell et al., 2002; Frawley et al., 2016). These preliminary findings are useful to inform future research for such populations. Replicating the study with a control group to evaluate the effects on migraine frequency should be possible since Vipassana meditation retreats exist all around the world. Recruitment targets could be challenging at a single center but would be more feasible if multiple centers were used. A trial including a placebo arm would be ideal, however, creating a 10-day placebo arm could be logistically difficult. Furthermore, since behavioral placebo arms can be very effective, the nature of such a placebo arm would need to be carefully planned. A randomized trial with a wait list arm should be feasible as a next step.

## Supplementary Material

supplemental

## Figures and Tables

**Table 1:** Baseline characteristics

Variable, mean (sd)	CM (*n* = 36)	EM (*n* = 9)	NC (*n* = 13)
Age	47.1 (12.9)	46.0 (12.8)	40.7 (12.1)
Sex (% female)	89	78	77
Race (% white nonhispanic)	86	78	100
Education (% bachelors or above)	89	44	54
Annual income			
% less than US$ 50 k	19	44	23
%50–100 k	36	0	31
% > 100 k	44	56	46
AUDIT score^a^	1.5 (2.0)	1.6 (1.5)	1.4 (1.0)
Yrs since migraines started	20.9 (14.1)	11.0 (4.6)	11.7 (11.4)
MIDAS^a^	62.7 (53.3)	22.3 (15.6)	120.7 (83.9)
MIDAS-6^a^ (# HA d/3 mos)	70.9 (22.1)	21.4 (14.2)	65 (35.7)
MIDAS-7^a^ (avg pain/3 mos)	5 (2)	5.6 (1.4)	7.1 (0.9)
MSQ			
MSQ Restrictive^b^ (limited by migraine)	41.2 (17.6)	55.9 (29.0)	31.0 (23.2)
MSQ Preventive^b^ (interrupted by migraine)	60.3 (20.7)	75.6 (17.4)	45.8 (25.2)
MSQ Emotional^b^ (frustration/helplessness)	46.1 (23.7)	57.0 (29.1)	31.8 (22.6)
Pain catastophizing^a^	22.9 (11.6)	22.4 (13.2)	25.8 (7.5)
GHQ 28^a^	25.6 (11.7)	24.7 (13.0)	31.2 (12.3)
PSS (4 item)^a^	6.7 (2.6)	7.2 (2.3)	7.6 (3.4)
PANAS negative^a^	19.7 (7.8)	27.6 (12.2)	25.5 (10.0)
PANAS positive^b^	27.5 (7.9)	32.2 (7.3)	24.5 (10.1)
Mindfulness^b^	35.1 (6.7)	33.9 (7.0)	36.2 (8.7)

*CM*, chronic migraine; *EM*, episodic migraine; *NC*, Noncompleter (dropped out of 10-day course after starting it); *AUDIT*, Alcohol Use Disorders Identification Test; *MIDAS*, Migraine Disability Assessment Test; *MIDAS-7* asks about average pain on 0–10 scale; *MSQ*, Migraine Specific Quality of Life Questionnaire domains: Restrictive dimension examines the degree to which performance of daily activities is limited by migraine, Preventive dimension examines the degree to which performance of daily activities is interrupted by migraine, Emotional dimension examines feelings of frustration and helplessness due to migraine; *GHQ 28*, General Health Questionnaire 28; *PSS*, Perceived Stress Scale; *PANAS*, Positive and Negative Affect Scale; *Mindfulness*, Freiburg Mindfulness Inventory; *mos*, months

a Lower score = improved health/scale outcome

b Higher score = improved health/scale outcome

**Table 2:** Mean results from daily headache diaries for chronic and episodic migraine (*n* = 45)

	PRE	POST	Difference [95% CI]^a^
Migraine days/28 d	16.6 d	13.8 d	− 2.7 [− 4.3, − 1.3]
Headache days/28 d	20.1 d	16.7 d	− 3.4 [− 4.9, − 1.9]
50% responder rate @ 12 months (migraine)^b^	–	29%	
Headache intensity (0–10 scale)	3.9	3.3	− 0.7 [− 0.9, − 0.4]
Headache length (hrs)	2.0	1.8	− 0.2 [− 0.4, + 0.0]
Missed work (days/28 d)	9.9 d	7.8 d	− 2.1 [− 4.6, + 0.4]
reduced activity (days/28 d)	21.9	19.6	− 2.3 [− 4.0, − 0.5]
Sleep duration (hrs)	6	6	0.09 [− 0.0, + 0.2]
Sleep quality (0–10 scale)	5	5	0.3 [0.1, 0.6]
Acute medication use/28 d	13.1	10.9	− 2.2 [− 3.9, − 0.5]

*d*, days

a Obtained from generalized estimating equation regression. Therefore, differences may not be exactly what is obtained by subtracting the crude rates

b For the period 9–12 months post-retreat

**Table 3:** Mean changes in secondary outcomes from baseline (*p* value); *n* = 45

	Baseline	Post 10-day retreat	3 months	6 months	12 months
MIDAS^a^	55.2	–	− 20.2 (0.05)	− 13.2 (0.07)	− 22.3 (< 0.01)
MIDAS6^a^ (#HA days/3mos)	58.4	–	− 11.1 (0.04)	− 9.2 (0.04)	− 16.9 (< 0.01)
MIDAS7^a^ (avg pain/3mos)	5.3	–	− 0.8 (0.03)	− 0.2 (0.46)	− 0.6 (0.02)
MSQ					
Restrictive^b^	48.2	19.3 (< 0.01)	15.7 (< 0.01)	14.2 (< 0.01)	20.3 (< 0.01)
Preventive^b^	66.4	16.1 (< 0.01)	10.1 (0.01)	11.2 (< 0.01)	12.3 (< 0.01)
Emotional^b^	51.7	24.5 (< 0.01)	18.2 (< 0.01)	17.1 (< 0.01)	22.5 (< 0.01)
Pain catastrophizing^a^	21.0	− 7.6 (< 0.01)	− 8.5 (< 0.01)	− 8.0 (< 0.01)	− 10.3 (< 0.01)
GHQ 28^a^	24.3	− 9.6 (< 0.01)	− 5.0 (0.04)	− 2.6 (0.23)	− 7.2 (< 0.01)
PSS (4 item)^a^	6.7	− 1.7 (< 0.01)	− 1.1 (0.03)	− 1.3 (< 0.01)	− 1.5 (< 0.01)
PANAS, negative^a^	21.4	− 5.2 (< 0.01)	− 2.6 (0.07)	− 1.4 (0.35)	− 4.2 (< 0.01)
PANAS, positive^b^	29.2	3.9 (< 0.01)	2.6 (0.02)	1.5 (0.27)	3.7 (< 0.01)
Mindfulness^b^	35.5	3.8 (< 0.01)	2.4 (0.01)	2.3 (0.05)	3.7 (0.01)

changes and *p*-values obtained from generalized estimating equations; *MIDAS*, Migraine Disability Assessment Test; *MIDAS-7* asks about average pain on 0–10 scale; *MSQ*, Migraine Specific Quality of Life Questionnaire domains: Restrictive dimension examines the degree to which performance of daily activities is limited by migraine, Preventive dimension examines the degree to which performance of daily activities is interrupted by migraine, Emotional dimension examines feelings of frustration and helplessness due to migraine; *GHQ 28*, General Health Questionnaire 28; *PSS*, Perceived Stress Scale; *PANAS*, Positive and Negative Affect Scale; *Mindfulness*, Freiburg Mindfulness Inventory; *mos*, months

a Lower score = improved health/scale outcome

b Higher score = improved health/scale outcome

## Data Availability

The dataset analyzed in his article is not publicly available.
